# Supplementary material for: The study of the characteristics of the secondary flowering of Cerasus subhirtella ‘Autumnalis’
Source: PeerJ. 2023 Mar 6;11:e14655. doi: 10.7717/peerj.14655 (PMC9997188; doi:10.7717/peerj.14655)
Supplement: Supplemental Information 4 [file peerj-11-14655-s004.docx]

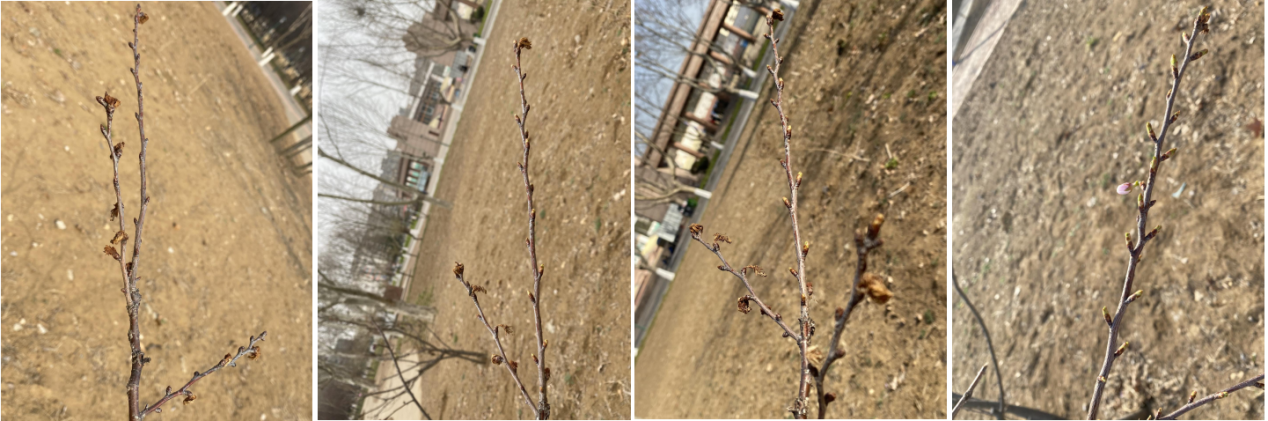


1. subhirtella ‘Autumnalis’ leaf bud development


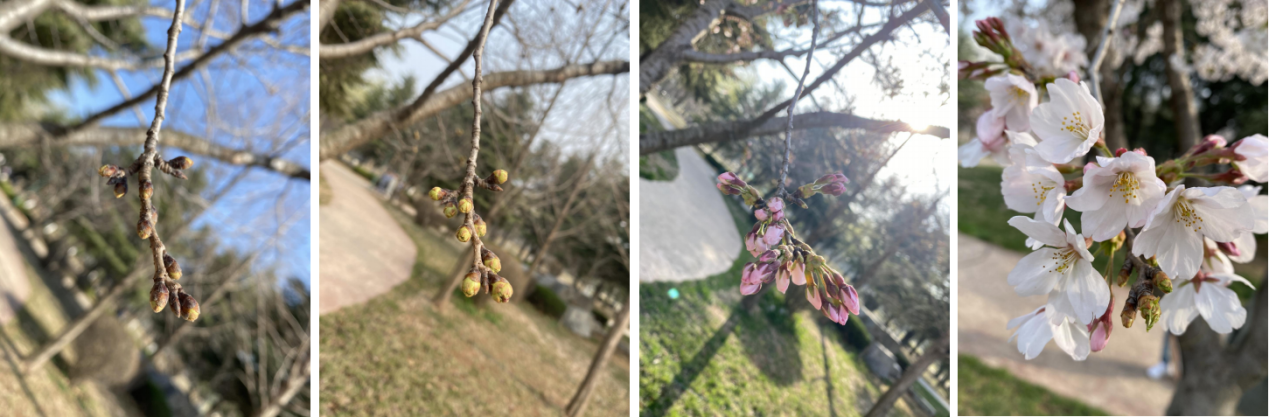


C. yedoensis ‘Somei yoshino’ leaf bud development


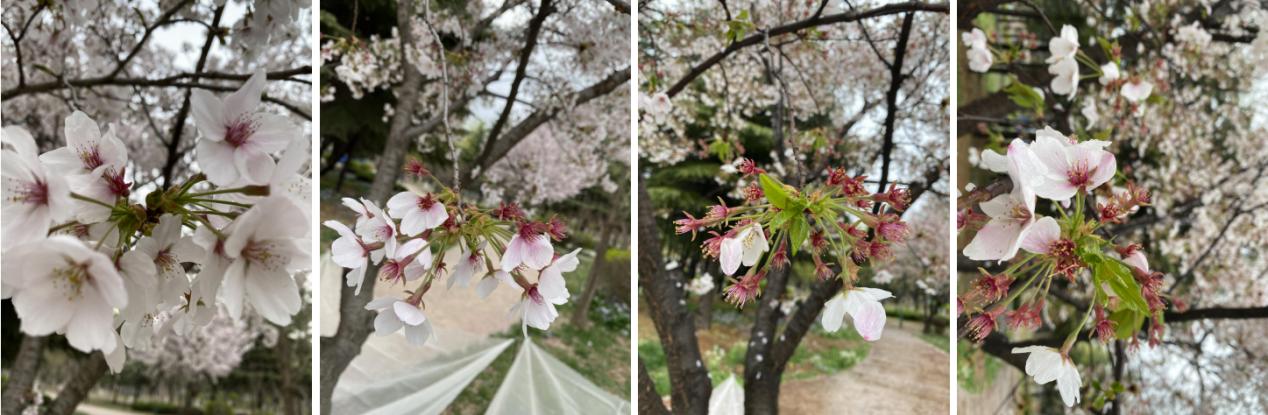


C. yedoensis ‘Somei yoshino’ leaf development


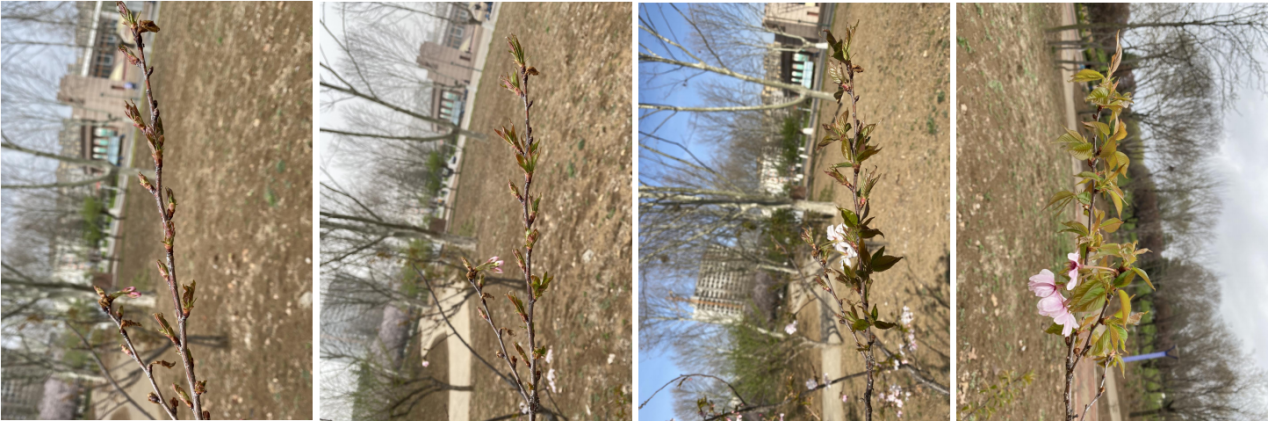


C. subhirtella ‘Autumnalis’ leaf development


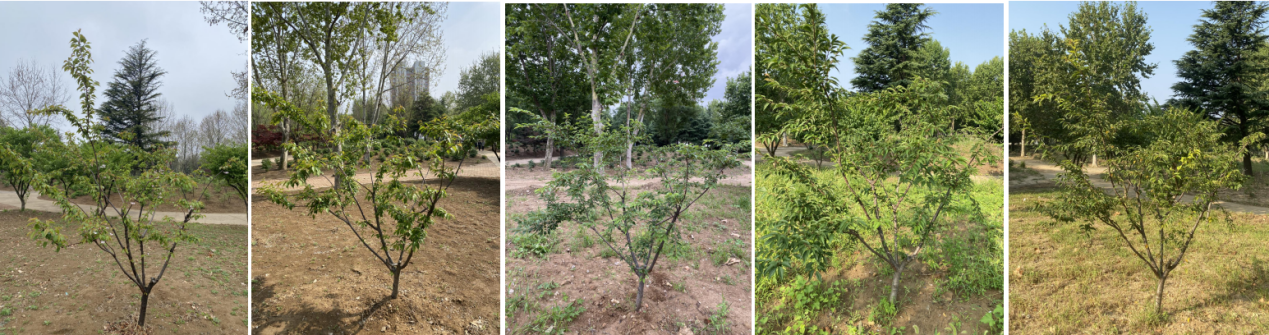
C. subhirtella ‘Autumnalis’ shoot development


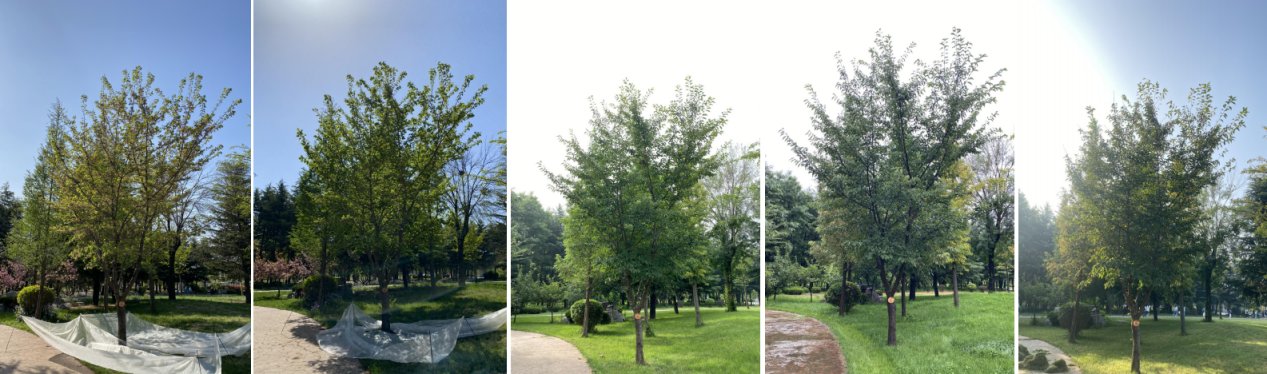


1. yedoensis ‘Somei yoshino’ Shoot development


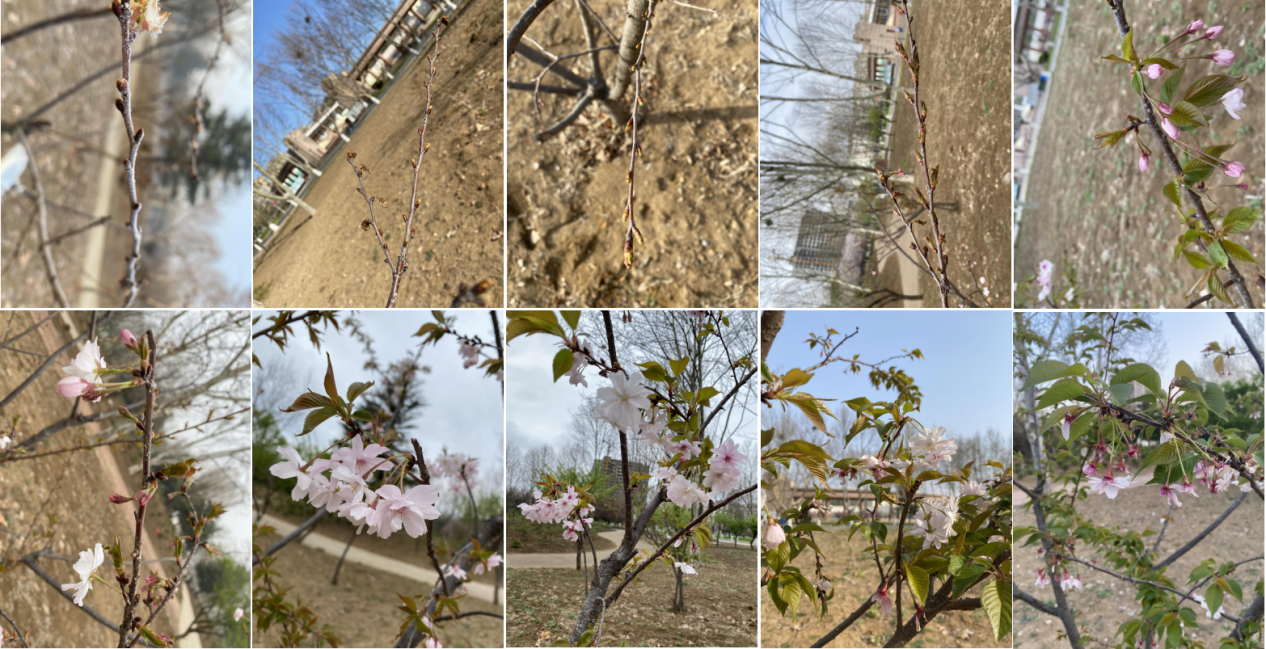


C. subhirtella ‘Autumnalis’ flower development (spring flowering period)


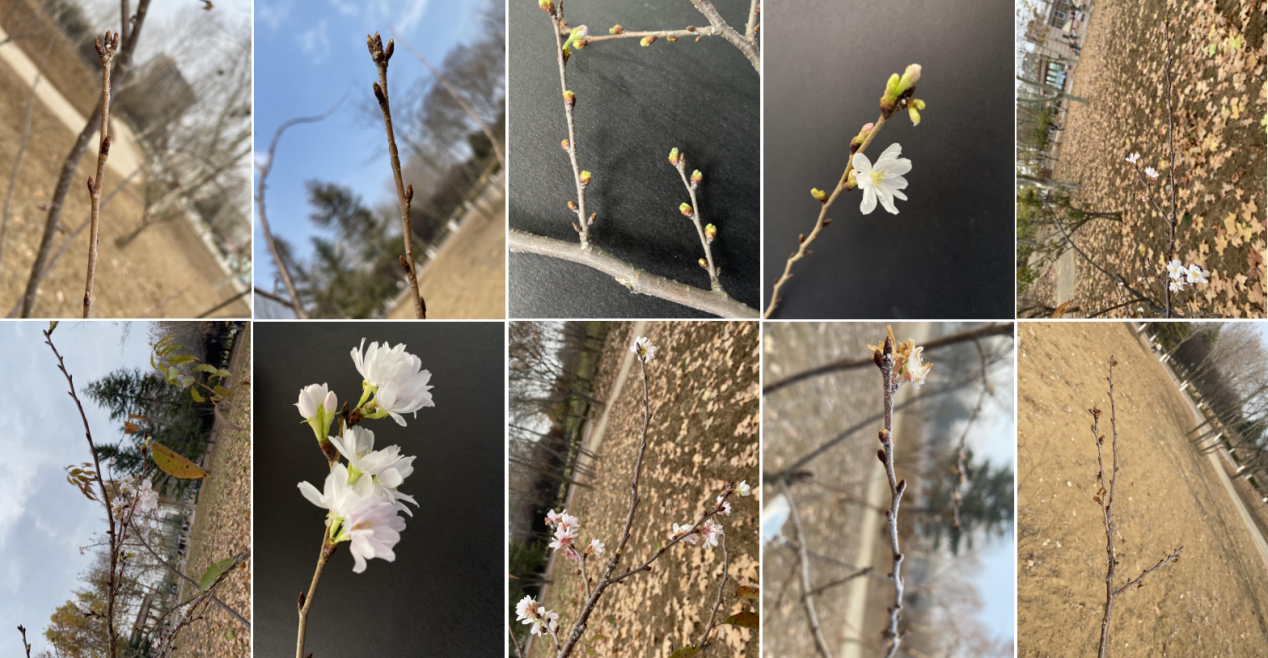


C. subhirtella ‘Autumnalis’ flower development (autumn flowering period)


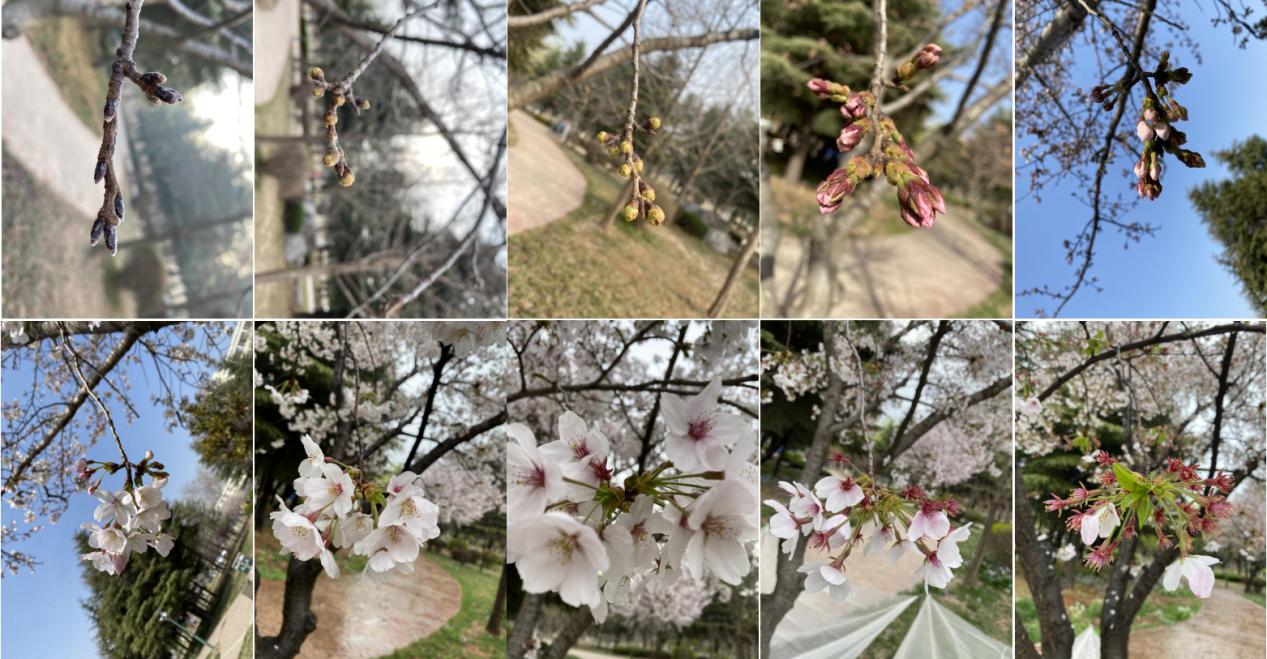


C. yedoensis ‘Somei yoshino’ flower development


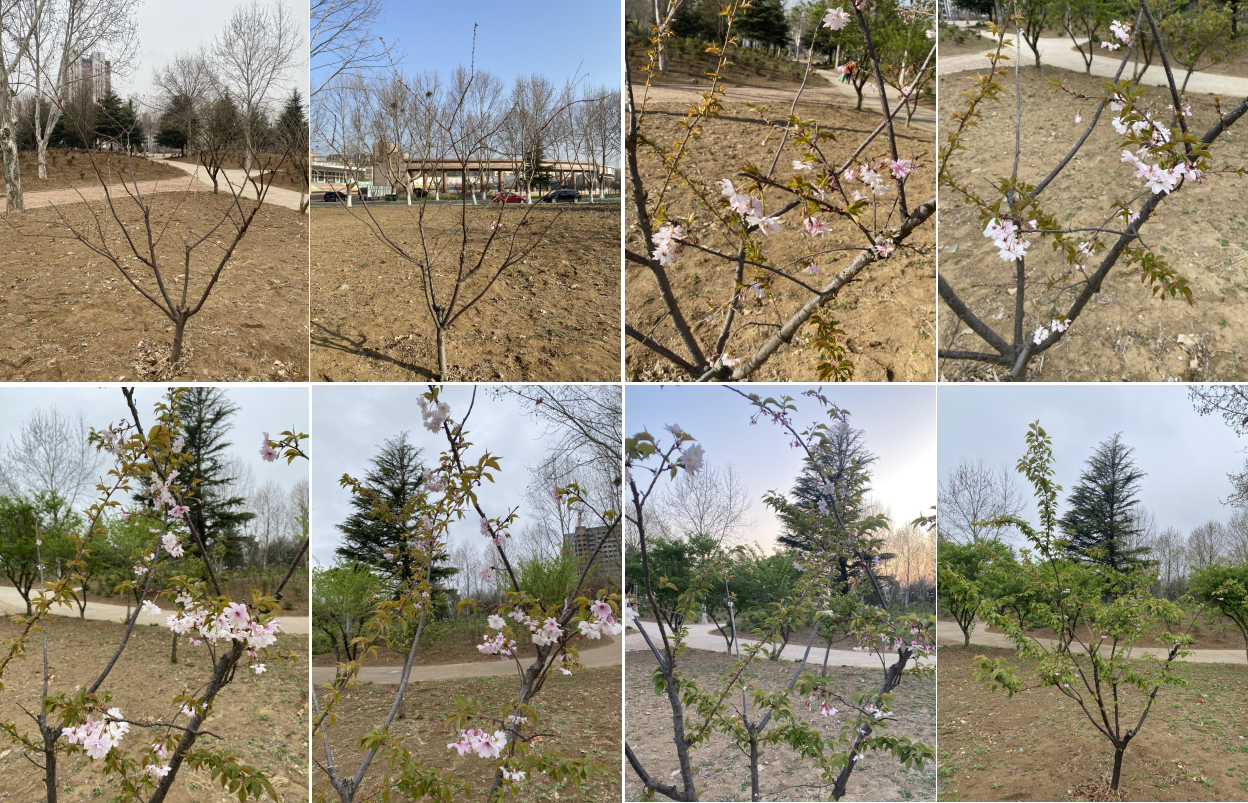


C. subhirtella ‘Autumnalis’ flower development


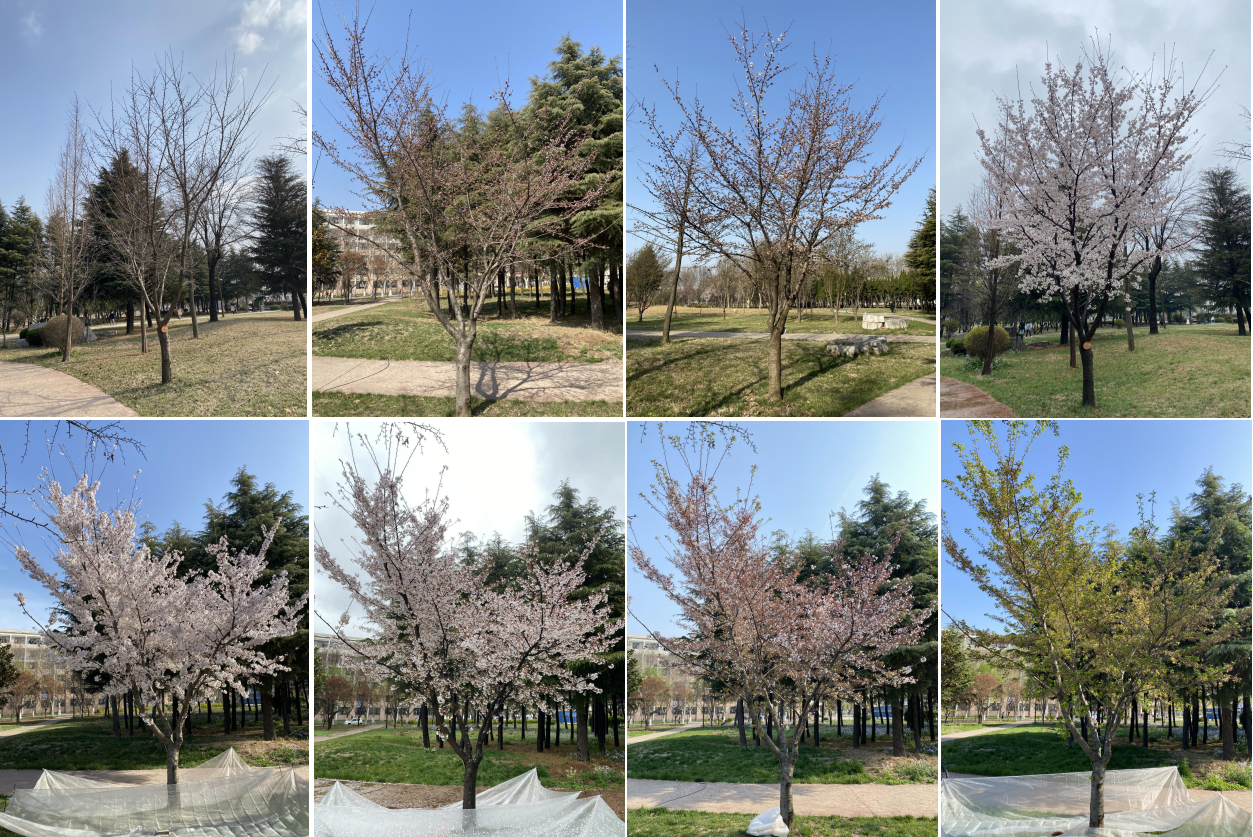


C. yedoensis ‘Somei yoshino’ flower development


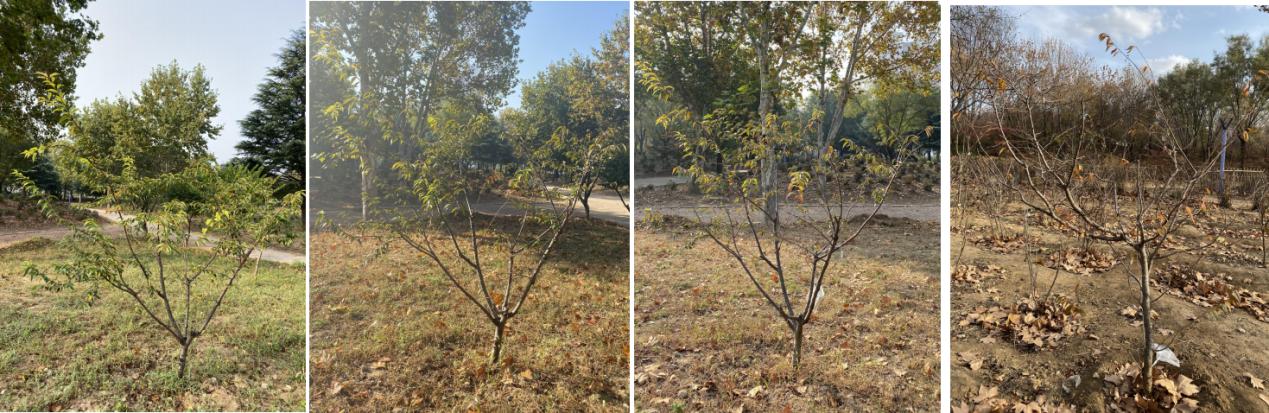


C. subhirtella ‘Autumnalis’ leaves fall, dormancy begins


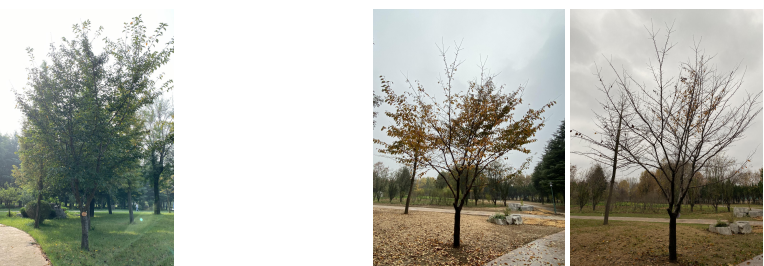


C. yedoensis ‘Somei yoshino’ leaves fall, dormancy begins
