# Supplementary material for: The study of the characteristics of the secondary flowering of Cerasus subhirtella ‘Autumnalis’
Source: PeerJ. 2023 Mar 6;11:e14655. doi: 10.7717/peerj.14655 (PMC9997188; doi:10.7717/peerj.14655)
Supplement: Supplemental Information 5 [file peerj-11-14655-s005.docx]

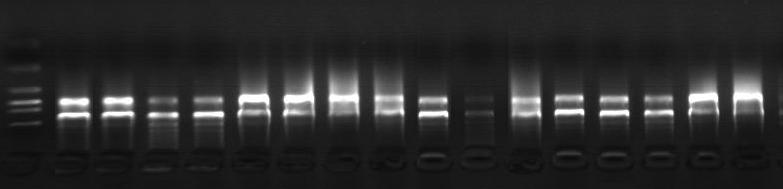

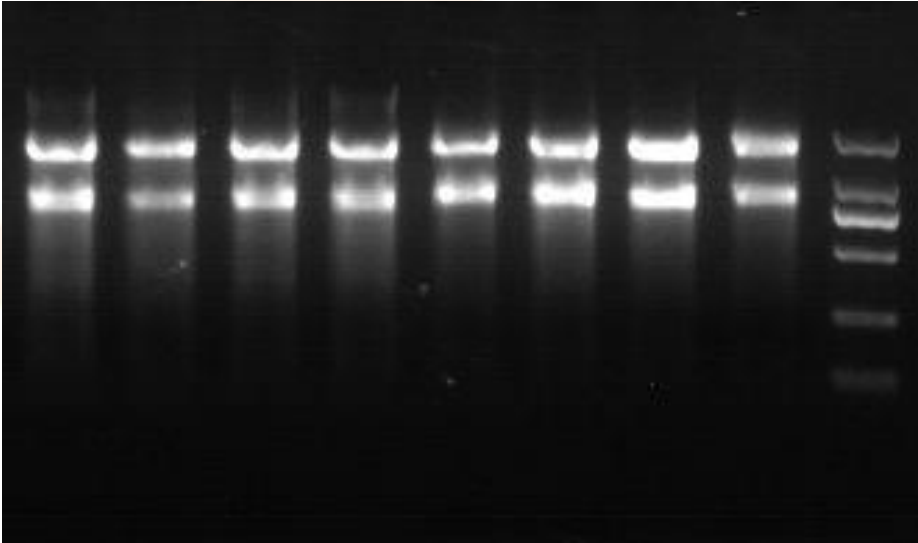

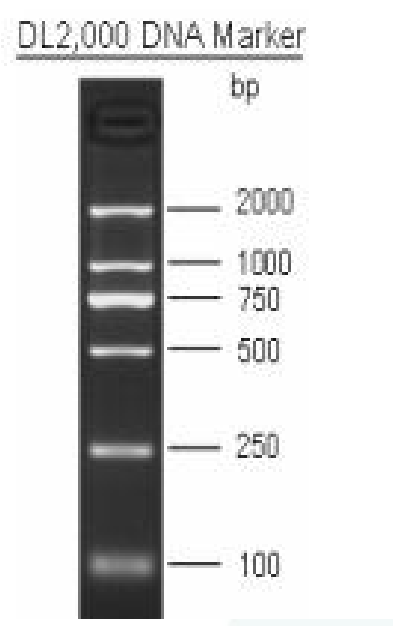


Detection of RNA by electrophoresis The complete RNA electropherogram generally has 3 bands, from top to bottom are 28S, 18S and 5S rRNA. The purpose of electrophoresis is to detect the integrity of 28S and 18S. If the band is clear, single, and bright, the RNA is considered good.
